# Nuclear Heme Oxidase-1 Inhibits Endoplasmic Reticulum Stress-Mediated Apoptosis after Spinal Cord Injury

**DOI:** 10.1155/2020/7576063

**Published:** 2020-08-02

**Authors:** Yunlong Bi, Xi Chen, Yang Cao, Deshui Yu, Jia'ai Zhao, Yu Jing, Gang Lv

**Affiliations:** ^1^Department of Orthopaedics, The First Affiliated Hospital of China Medical University, Shenyang 110001, China; ^2^Department of Orthopaedics, The First Affiliated Hospital of Jinzhou Medical University, Jinzhou 121000, China; ^3^Department of Orthopaedics, The Third Affiliated Hospital of Jinzhou Medical University, Jinzhou 121000, China; ^4^Department of Neurology, The First Affiliated Hospital of Jinzhou Medical University, Jinzhou 121000, China; ^5^Department of Oncology, The First Affiliated Hospital of Jinzhou Medical University, Jinzhou 121000, China

## Abstract

The treatment goal for spinal cord injury (SCI) is to repair neurites and suppress cellular apoptosis. This study is to investigate the effects of nuclear heme oxidase-1 (HO-1) on the acute spinal cord injury and the related mechanisms. The rat model of the SCI was established. On day 7, before model establishment, the adenovirus vector carrying nuclear HO-1 (Ad-GFP-HO-1C*Δ*23) was injected into the animals into the tenth thoracic spine (T10) segment by the intrathecal injection. Starting from after the model establishment to day 28, the recovery of motor function was assessed by the Basso-Beattie-Bresnahan (BBB) scoring method. Immunofluorescence was performed to detect the expression patterns of nuclear and cytoplasmic proteins. HE and Nissl staining methods were used to evaluate the structural damage and the number of surviving neurons near the injured area. The TUNEL method was conducted to evaluate the apoptotic degree. Protein expression levels were detected with the Western blot analysis. The BBB assay scores in the nuclear HO-1 group were significantly higher than the blank and adenovirus control groups. Moreover, compared to the blank and adenovirus control groups, the neuronal apoptosis in the nuclear HO-1 group was significantly alleviated. Furthermore, the expression levels of the endoplasmic reticulum stress-related proteins, i.e., CHOP, GRP78, and caspase-12, were significantly decreased in the nuclear HO-1 group. Nuclear HO-1 significantly improves the SCI, promotes the functional recovery, inhibits the endoplasmic reticulum stress, and alleviates the apoptotic process after SCI.

## 1. Introduction

Spinal cord injury (SCI) is a common traumatic disease with an extremely high disability rate. The pathology of SCI could be divided into two stages, i.e., the primary damage caused by direct injury to the spinal cord tissue and the secondary damage including the axon destruction, neuronal death, inflammation, blood-brain barrier destruction, hypoxia, and ischemia [1]. These events during the secondary injuries would always lead to the neuronal death and dysfunction. Therefore, the treatment of SCI mainly focuses on the secondary injuries, through reducing the neuronal death and repairing the neural circuit function. Recent reports have shown that the endoplasmic reticulum stress (ER stress) response is activated after the moderate contusion of the spinal cord in rodent models, and the inhibition of endoplasmic reticulum stress can improve the recovery of the hindlimb motor function [2, 3].

ER stress is one of the key pathological changes that lead to cell death and neuronal dysfunction after SCI. In the second stage of SCI, excessive ER stress could be seen in the areas near the injury site. The accumulation of unfolded proteins within ER would initiate the unfolded protein response (UPR) [4]. Therefore, UPR can be used as an indicator for ER stress. The UPR could be activated by three signal pathways, involving the IRE1, ATF6, and PERK, respectively [5]. During this process, the expression levels of apoptotic proteins are increased, further leading to neuronal apoptosis [6]. It has been shown that the UPR-related molecules (such as GRP78 and CHOP) would be upregulated at the early stage after SCI. In addition, under the condition of severe ER stress, the UPR would eventually trigger the apoptotic pathways, and one of the important molecules related to cell death is caspase-12 [7, 8]. Heme oxidase-1 (HO-1) is a cytoprotective enzyme encoded by the HMOX1 gene (gene ID: 396287). The induction of HO-1 may be an important event in certain acute reactions and cytoprotection after injury [9]. Further studies have confirmed that the nuclear HO-1 enhances the cell's protection against the oxidative stress, and the transfection of nuclear C23 constructs could increase the cell survival [10, 11].

In this study, the role of nuclear HO-1 in the recovery process of SCI after SCI was investigated. The association of nuclear HO-1 with the endoplasmic reticulum (ER) stress-related proteins was studied, and the possible mechanism was also analyzed.

## 2. Materials and Methods

### 2.1. Preparation of Ad-GFP-HO-1C*Δ*23

To prepare the Ad-GFP-HO-1C*Δ*23, the seamless cloning technology was used, with the following primer: r-HO-1C*Δ*23-Eco/Eco-Fg, gtgaccggcgcctacgccaccATGGAGCGCCCACAGCTC and r-HO-1C*Δ*23-Eco/Eco-Rg, ggatcccgcccggggTGTCTGGGATGAACTAGT. The target gene HO-1C*Δ*23 fragment (5′-TTCAGATCTATGGAGCGCCCACAGCAC-3′) was ligated with the adenoviral vector pHBAD-EF1-MCS-3flag-CMV-GFP (synthesized by the Hanheng Biotechnology Co., Ltd., Shanghai, China). The Ad-GFP and Ad-GFP-HO-1C*Δ*23 were amplified in the HEK293 cells and purified and stored until further use.

### 2.2. Study Animals

The experimental rats were randomly divided into four groups (*n* = 8 per group): the (1) sham (sham), (2) blank (SCI), (3) adenovirus control (Ad-GFP), and (4) adenovirus-mediated nuclear HO-1 (Ad-HO-1) groups. All animal experiments were performed in accordance with the Guidelines for the National Laboratory Animal Care and Use Health, and were approved by the Animal Ethics Committee of Jinzhou Medical University. At day 7 before the SCI model establishment, the rats were intraperitoneally injected with 2% sodium pentobarbital (Sigma, St Louis, MO, USA), the rats from the sham and blank groups were subjected to the injection of 5 *μ*l PBS (0.01 mol/l) into the spinal cord T10 segment through a microsyringe [12]. On the other hand, for the adenovirus control and adenovirus-mediated nuclear HO-1 groups, the rats were injected with 5 *μ*l Ad-GFP (6.3 × 10^8^ PFUs/5 *μ*) and Ad-GFP-HO-1C*Δ*23 (7.9 × 10^8^ PFUs/5 *μ*), respectively, into the spinal cord T10 segment [13].

### 2.3. SCI Model Establishment

For the model establishment, the rats were anesthetized with the intraperitoneal injection of 2% sodium pentobarbital (Sigma), at a dose of 30 mg/kg. An incision was made at the T10 vertebral body of the spinal cord, and the skin and subcutaneous tissues were cut open. The paravertebral muscles were separated to expose the lamina, and the T10 segment laminectomy was performed to fully expose the spinal cord tissue. The model establishment was conducted using the modified Allen's striking method with a spinal cord punch. Centering on the T10 segment, a 20 g weight freely fell from a height of 30.0 mm, during which process the membrane integrity should be maintained. The tail spasticity, as well as the lower limb tremor and flaccid paralysis, indicated the successful establishment of the rat SCI model. For the sham group, only the T10 thoracic spine was removed without any other operations [14].

### 2.4. Immunofluorescence Staining

Sections were infiltrated with PBS containing 0.5% Triton X-100. After blocking with 10% goat serum at room temperature for 1 h, the section was incubated with the primary antibodies at 4°C overnight. After washing with 0.1% PBST, the sections were incubated with the secondary antibody at room temperature for 2 h. After another round of washing with 0.1% PBST, the section was treated with DAPI. After glycerol mounting, the fluorescence was observed under a fluorescence microscope.

### 2.5. Animal Behavior Performance Assessment

On days 1, 3, 7, 14, 21, and 28 after surgery, respectively, the hindlimb motor function of the rat models was assessed with the Basso-Beattie-Bresnahan (BBB) exercise scores, including totally 21 hindlimb exercise standards [15]. The scoring was based on the accurate observations of the hindlimb gait, joint motion, and coordination by observers who were unaware of the experimental conditions.

### 2.6. Western Blot Analysis

Total protein was extracted from the spinal cord with the RIPA lysis. After concentration determination, the protein sample was separated with 10% SDS-PAGE gel. The protein was electronically transferred onto a PDVF membrane. After blocking with 5% skim milk at 4°C for 2 h, the membrane was incubated with the primary antibody at 4°C overnight. After washing with TBST, the membrane was incubated with the secondary antibody at room temperature for 2 h. Color development was performed, and the protein bands were imaged and analyzed with the ImageJ software.

### 2.7. Hematoxylin-Eosin (H) and Cresol Purple (Nissl) Staining

To evaluate the structural damages and neuronal survival near the injured area after surgery, HE and Nissl staining was performed. The animals were sacrificed at day 7 after surgery, the T8-T11 tissues were removed, followed by the OTC embedding. The tissues were cut into 10 *μ*m sections, which were subjected to the HE and Nissl staining, according to the manufacturer's instructions.

### 2.8. Terminal Deoxynucleotidyl Transferase dUTP Nick-End Labeling (TUNEL)

Damaged DNA fragments were stained using the one-step TUNEL apoptosis detection kit (Beyotime, Haimen, Jiangsu, China). The apoptotic neurons would show red fluorescence, and the ratio of the red fluorescent area to the total area was used to calculate the percentage of apoptotic cells. Quantitative analysis was performed by counting the number of TUNEL-positive cells in three sections for each rat.

### 2.9. Statistical Analysis

All values were expressed as mean ± SD. Data were evaluated by one-way analysis of variance, followed by Tukey's multiple comparison test. Statistical analysis was performed with the SPSS 23.0 software (SPSS Inc., Chicago, Illinois, USA). *P* < 0.05 was considered statistically significant.

## 3. Results

### 3.1. HO-1 Nuclear Translocation

**Figure 1:**
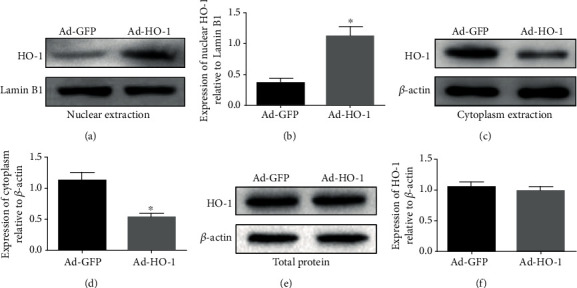
Nucleus and cytoplasmic HO-1 protein expressions. The HO-1 expression levels in the nuclear (a, b), cytoplasm (c, d), and whole cells e, f) were detected with the Western blot analysis, respectively. Values were presented as mean ± SD (*n* = 3). Statistical analysis was performed by one-way analysis of variance, followed by Tukey's multiple comparison test. ^∗^*P* < 0.05 vs. the Ad-GFP group.

**Figure 2:**
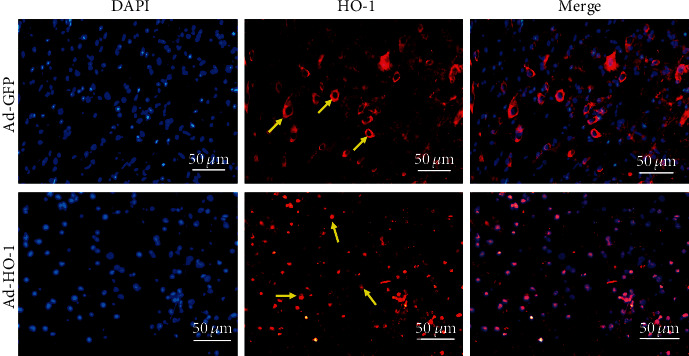
Nuclear translocation of HO-1. The nuclear translocation of HO-1 in the Ad-GFP and Ad-HO-1 groups was observed under a fluorescence microscope. The yellow arrows denote HO-1. The images were obtained at low microscope objective magnification of 20x.

To investigate the expression levels of HO-1 in the nuclear, the Western blot analysis was performed. As shown in Figures 1(a) and 1(b), our results indicated that the nuclear HO-1 protein expression levels in the Ad-nuclear HO-1 group were significantly higher (3.08 times) than the Ad-GFP negative control group. Moreover, the expression levels of cytoplasm HO-1 in the Ad-GFP group were significantly higher (2.1 times) than the Ad-HO-1 group (Figures 1(c) and 1(d)). There was no significant difference in total HO-1 expression in whole cells between these two groups (Figures 1(e) and 1(f)). In addition, as shown in Figure 2, our results from the immunofluorescence staining indicated that, compared with the Ad-GFP group, obvious HO-1 nuclear translocation was observed in the Ad-HO-1 group.

### 3.2. Nuclear HO-1 Promotes Motor Recovery after SCI and Reduces Structural Damages and Neuronal Necrosis

**Figure 3:**
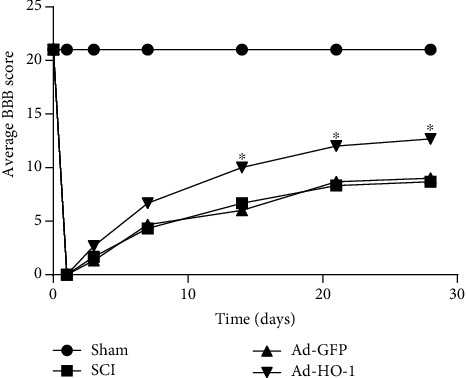
Recovery of motor function assessment. The recovery of the motor function was assessed with the BBB scoring method. Values were presented as mean ± SD (*n* = 3). Statistical analysis was performed by one-way analysis of variance, followed by Tukey's multiple comparison test. ^∗^*P* < 0.05 vs. the Ad-GFP group.

The recovery of motor function was assessed by the Basso-Beattie-Bresnahan (BBB) scoring. Our results showed that there were no significant difference in the BBB scores between the SCI group and the Ad-GFP group (Figure 3). However, the BBB score for the Ad-HO-1 group was significantly higher than that for the SCI group and the Ad-GFP group, suggesting that the nuclear HO-1 would improve the exercise recovery after SCI.

**Figure 4:**
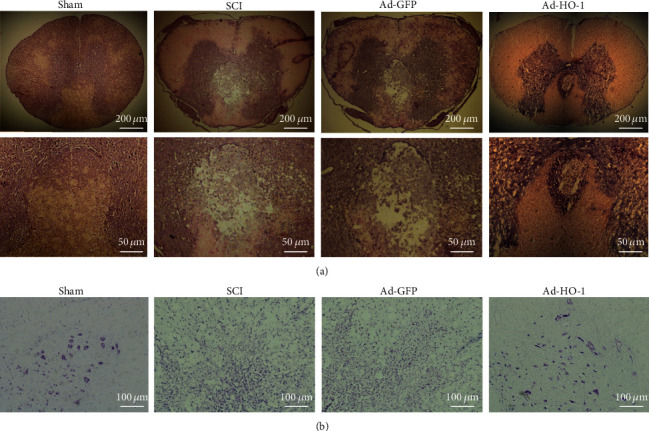
Observation of structural damages and neuronal necrosis. (a, b) The structural damages and neuronal necrosis were detected with the HE staining (a) and Nissl staining (b). The images were obtained at low microscope objective magnification of 5x, 20x, and 10x.

On the other hand, as shown in Figure 4(a), our results from the histochemical staining indicated that significant structural damages were observed in the SCI group and the Ad-GFP group. In contrast, the Ad-HO-1 group exhibited less necrosis near the injury. As shown in Figure 4(b), our results from the Nissl staining indicated that, at 4 weeks after surgery, compared with the SCI group and the Ad-GFP group, the neuronal loss in the anterior horn was significantly less in the Ad-HO-1 group.

### 3.3. Nuclear HO-1 Inhibits Cellular Apoptosis after SCI

**Figure 5:**
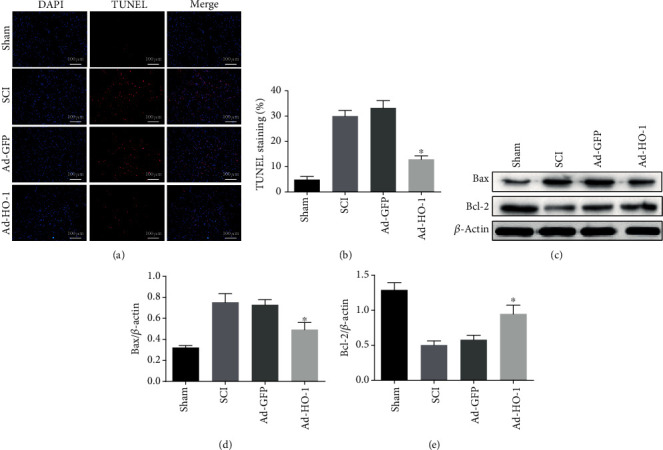
Analysis of neuronal apoptosis. (a, b) The neuronal apoptosis was detected with the TUNEL (a) and the statistical analysis (b). The images were obtained at low microscope objective magnification of 20x. (c–e) The apoptosis-related protein expression levels were detected with the Western blot analysis (c) and the statistical analysis of the expression levels of Bax (d) and Bcl-2 (e). Values were presented as mean ± SD (*n* = 3). Statistical analysis was performed by one-way analysis of variance, followed by Tukey's multiple comparison test. ^∗^*P* < 0.05 vs. the Ad-GFP group.

As shown in Figures 5(a) and 5(b), our results from the TUNEL assessment indicated that the apoptotic levels in the SCI group and Ad-GFP group were significantly high, which was significantly decreased in the Ad-HO-1 group. Moreover, as shown in Figures 5(c)–5(e), our results from the Western blot analysis indicated that the nuclear HO-1 reduced the expression levels of Bax after SCI and increased the expression of Bcl-2 (an antiapoptotic protein). These results suggest that the nuclear HO-1 could inhibit the cellular apoptosis after SCI.

### 3.4. Nuclear HO-1 Inhibits ER Stress after SCI

**Figure 6:**
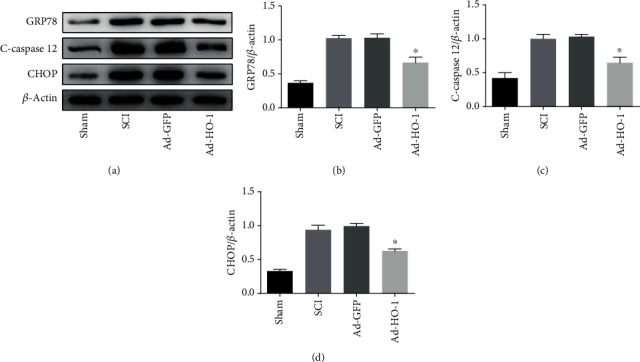
Analysis of ER stress-related protein expression. (a–d) The ER stress-related protein expression levels were detected with the Western blot analysis (a), and the statistical analysis of the expression levels of GRP78 (b), caspase-12 (c), and CHOP (d). Values were presented as mean ± SD (*n* = 3). Statistical analysis was performed by one-way analysis of variance, followed by Tukey's multiple comparison test. ^∗^*P* < 0.05 vs. the Ad-GFP group.

As shown in Figure 6 our results from the Western blot analysis indicated that the expression levels of ER stress-related proteins in the SCI group and the Ad-GFP group were high, while the related protein expression levels in the Ad-HO-1 group were significantly decreased compared with the former two groups. These results suggest that the nuclear HO-1 could inhibit the ER stress after SCI.

## 4. Discussion

In the present study, the role of nuclear HO-1 in inhibiting the neuronal apoptosis after SCI was investigated, as well as the possibly related mechanisms. Our results showed that the nuclear HO-1 significantly increased the Bcl-2/Bax ratio and inhibited the expression levels of CHOP, caspase-12, and GRP78. These results suggest that the neuroprotective effects of nuclear HO-1 on SCI may be achieved, at least partially, by inhibiting the ER stress-induced apoptosis pathways.

SCI consists of primary and secondary injuries. Primary injuries are limited to the vertebral fracture area, which are characterized by acute bleeding and ischemia. Secondary injuries involve a series of complex molecular events that might lead to the ion disturbance, further resulting in the local edema, focal bleeding, oxidative stress, inflammation, and extensive neuronal death. Further destruction of neurons and glial cell could induce the significant damage expansion, probably causing paralysis extension to higher segments [2]. After SCI, cell death occurs at the injury site, including the posttraumatic necrosis and apoptosis. Apoptosis is a highly regulated mode of cell death, which is important for the normal development and homeostasis. However, excessive or deficient apoptosis could also lead to various disorders [16].

Heme oxygenase (HO) is the rate-limiting enzyme in the heme catabolism. So far, three different subtypes of mammalian HO have been reported, i.e., HO-1, HO-2, and HO-3, with different tissue-specific gene expression patterns [17]. HO-1 is an inducible, ubiquitous 32 kDa subtype [18]. The induction of HO-1 would clear the potentially toxic prooxidant molecule heme and produces the metabolite bilirubin with antioxidant properties. Enhancement of the HO activity through the pharmacological or genetic methods could ease various pathological processes in human beings [9]. Studies have shown that the increased HO-1 could limit the oxidative disorders caused by ER-related protein misfolding and reduce the unfolded protein responses, thus decreasing the levels of ER stress markers such as the reactive oxygen species (ROS) [19, 20]. The ER stress would induce the HO-1 gene expression in the vascular smooth muscle cells, and the HO-1 could catalyze the process of CO inhibiting ER stress-mediated apoptosis. This apoptotic event is triggered by the activation of the proapoptotic transcription factor CHOP and the activation of ER-related caspase-12. The HO-1/CO system could serve as an important adaptive mechanism for ER stress to promote cell survival [9, 19, 20]. Although HO-1 is an intact protein of the smooth ER [21, 22], it could be located in other regions, including the pits [23], mitochondria [24], and even nuclei [10, 25], mediating the signaling transduction [26]. The negative results based on the antibody against the C-terminus identified this isomer as a 28 kDa HO-1 fragment, with 52 amino acids deleted at the C-terminus [10]. Nuclear HO-1 is found to be enzymatically inactivated, which however is associated with the enhanced activation of the transcription factor AP-1 [27]. A previous study has shown that the mutant, enzyme-inactivated HO-1 could still protect against the oxidative damages [28]. Further research has found that tnuclear HO-1 enhances the protection of cells against oxidative stress, and the transfection of C*Δ*23 constructs could improve cell survival [10, 11, 29]. Nuclear HO-1 interacts with the transcription factors such as AP-1 and Nrf2 to participate in regulating the antioxidant gene responses within cells under oxidative stress [10, 29]. The nuclear localization suggests that the HO-1 protein may have a signal transduction ability, which may regulate cell proliferation and upregulate the antioxidant defense. The nuclear localization of HO-1 is an important signaling pathway that provides cellular protection in the presence of oxidative stress [30]. During oxidative stress, nuclear HO-1 expression would be increased, leading to the transcriptional regulation of antioxidant enzymes and gaining a survival advantage [11].

Accumulation of unfolded and misfolded proteins in the ER has been called the ER stress. Cells address the protein folding defect by activating the unfolded protein response (UPR) [4]. UPR activation would help to maintain the protein homeostasis in the ER, responding to various cellular injuries, including hypoxia, ischemia, trauma, and oxidative damages. The signal cascade of ER stress involves three transmembrane proteins, i.e., the ER stress-activated protein kinase RNA- (PKR-) like kinase (PERK), inositol requirement protein-1a (IRE-1a) [31], and transcription factor 6 [5]. Under normal circumstances, the reaction series would successfully restore the dynamic balance of the ER. However, persistent or intense ER stress would trigger programmed cell death or apoptosis [6, 32–34]. Activation of ER stress after SCI leads to accumulation of unfolded proteins in the ER. Prolonging ER stress can inhibit protein synthesis and deplete the ER calcium ions, ultimately increasing the expression of CHOP and activating the caspase-dependent neuronal cell death [35]. The UPR-related molecules such as GRP78 and CHOP have been reported to be upregulated early after SCI. In addition, under severe ER stress, UPR eventually triggers the apoptotic pathway, and one of the important molecules related to cell death is caspase-12, which is considered to be a representative cell death signal [7, 8]. Under ER stress, the Bcl-2 expression would be inhibited by the CHOP expression. During ER stress, the Bax activation is a necessary link for neuronal death, which could eventually lead to neuronal apoptosis [36, 37]. ER stress-induced apoptosis is an important cause of neuronal death in many neurodegenerative diseases and after ischemia [38]. Therefore, based on these findings, inhibiting the ER stress after SCI may be an effective treatment strategy to inhibit neuronal apoptosis.

## 5. Conclusions

In conclusion, nuclear HO-1 can inhibit the progress of SCI and promote functional recovery after injury. The protective mechanism of nuclear HO-1 on SCI may be partially achieved by inhibiting the endoplasmic reticulum stress-induced apoptosis pathway. Nuclear HO-1 may become a new therapeutic target for promoting the recovery of SCI and reducing secondary SCI. Our findings are of great guiding significance for the treatment of SCI.

## Data Availability

The data used to support the findings of this study are included within the article.
